# Impact of Baseline Nutritional Status, Psychological Health, Fatigue, and Insomnia on Outcomes of Immune Checkpoint Inhibitors in Advanced Non‐Small Cell Lung Cancer: A Retrospective Cohort Study

**DOI:** 10.1002/kjm2.70097

**Published:** 2025-08-24

**Authors:** Yu‐Xuan Zhu, Yuan‐Yuan Zhang, Xiu‐Juan Jiang

**Affiliations:** ^1^ Geriatric Oncology Department Nanjing Drum Tower Hospital Nanjing China; ^2^ Department of Interventional Radiology Jiangsu Cancer Hospital Nanjing China

**Keywords:** immunotherapy, non‐small cell lung cancer (NSCLC), nutritional status, prognostic factors, psychological health

## Abstract

This study investigated the impact of pretreatment nutritional status, psychological health, fatigue, and insomnia on outcomes of immune checkpoint inhibitors (ICIs) monotherapy in patients with advanced non‐small cell lung cancer (NSCLC). A total of 80 patients with stage IV NSCLC were enrolled. Baseline assessments included the Controlling Nutritional Status (CONUT) score, Herth Hope Index (HHI), Hospital Anxiety and Depression Scale (HADS), Brief Fatigue Inventory (BFI), and Athens Insomnia Scale (AIS). Response to ICImonotherapy, along with progression‐free survival (PFS) and overall survival (OS), was evaluated at week eight and through subsequent survival analyses. At week eight, partial response (PR), stable disease (SD), and progressive disease (PD) were observed in 31.8%, 33.0%, and 35.2% of patients, respectively. PD patients had significantly higher pretreatment CONUT scores, greater anxiety and depression, and more severe fatigue and insomnia than PR patients. Low nutritional risk was associated with improved OS and PFS. Higher HHI scores and lower HADS‐A/D, BFI, and AIS scores correlated with better survival outcomes. In multivariate analysis, anxiety was independently associated with PFS, and depression and fatigue independently predicted OS. Pretreatment nutritional status, psychological health, fatigue, and insomnia significantly influence immunotherapy response and survival in advanced NSCLC. These findings underscore the clinical importance of comprehensive baseline assessments to identify high‐risk patients who may benefit from targeted interventions before initiating immunotherapy. Addressing nutritional deficits, psychological distress, fatigue, and insomnia early could potentially enhance treatment response and improve survival outcomes, offering valuable insights for personalized cancer care strategies.

## 1 Introduction

Lung cancer remains the leading cause of cancer‐related mortality worldwide, accounting for 11.6% of all cancer cases, with non‐small cell lung cancer (NSCLC) representing approximately 85%–87% of all lung cancer diagnoses [1, 2]. Until recently, treatment options for advanced‐stage NSCLC primarily involved combination chemotherapy, providing a progression‐free survival (PFS) of 4–6 months and an overall survival (OS) of around 12–18 months [3]. The emergence of immune checkpoint inhibitors (ICIs) has transformed the therapeutic landscape, significantly improving outcomes for select patients [3, 4]. ICIs, such as pembrolizumab, tislelizumab, and atezolizumab, target the PD‐1/PD‐L1 pathway to enhance immune surveillance and anti‐tumor responses [5]. Treatment regimens are often guided by PD‐L1 expression levels, with patients exhibiting high tumor proportion scores (TPS ≥ 50%) receiving monotherapy and others requiring chemoimmunotherapy or dual ICI combinations [6]. Despite these advancements, clinical outcomes remain highly variable. While tumor PD‐L1 expression serves as a key biomarker, it is insufficient to fully predict responses. Emerging evidence suggests that patient‐specific factors, such as nutritional status and systemic inflammation, can significantly affect ICI efficacy, as shown by associations between low BMI or high PLR and poorer survival outcomes [7, 8]. Nutritional status, psychological health, and specific physical symptoms have gained attention for their potential roles in modulating immunotherapy outcomes. However, the mechanisms underlying these associations remain unclear, and their implications for clinical practice are underexplored.

Nutritional status, often assessed using the Controlling Nutritional Status (CONUT) score, is a critical determinant of immune competence [9]. Poor nutritional status has been associated with systemic inflammation, reduced lymphocyte activity, and impaired anti‐tumor immunity, all of which can compromise treatment efficacy. Poor nutritional status, for instance, has been linked to systemic inflammation, immune suppression, and adverse oncologic outcomes [10]. For example, high CONUT scores have been linked to shorter survival times in NSCLC patients undergoing surgical or medical treatment, suggesting its prognostic value extends to immunotherapy [11].

Psychological health, including anxiety, depression, and hope, has also emerged as a key modulator of immune function and cancer outcomes. Chronic psychological distress may dysregulate the hypothalamic–pituitary–adrenal axis, leading to elevated cortisol levels, increased systemic inflammation, and suppressed anti‐tumor immunity [12, 13]. Hope, on the other hand, is associated with better psychological resilience and may indirectly enhance immune responses and treatment adherence [14]. Understanding the interplay between these psychological factors and immunotherapy efficacy could provide valuable insights for developing holistic care strategies.

Additionally, specific symptoms such as cancer‐related fatigue and insomnia are highly prevalent in NSCLC patients and have been shown to negatively affect quality of life and functional status. Fatigue, driven by systemic inflammation and metabolic disruptions, may impair physical activity and immune recovery, while insomnia can disrupt circadian rhythms and immune homeostasis [15, 16]. These symptoms, though often overlooked, may serve as modifiable predictors of treatment outcomes.

While these factors have been investigated individually in various cancer settings, their combined impact on immunotherapy outcomes in advanced NSCLC remains underexplored. A comprehensive understanding of how baseline nutritional, psychological, and symptomatic factors influence PFS and OS could guide the development of targeted interventions, including nutritional optimization, psychosocial support, and symptom management, to improve patient outcomes.

This retrospective study aims to evaluate the relationships between baseline nutritional status, psychological health, fatigue, and insomnia with treatment response, PFS, and OS in advanced NSCLC patients treated with ICIs monotherapy. By leveraging validated assessment tools, this study seeks to identify actionable prognostic factors and inform a more personalized and holistic approach to NSCLC management. The findings may provide a basis for integrating supportive care interventions alongside immunotherapy to enhance both survival and quality of life for these patients.

## 2 Methods and Materials

### 2.1 Ethics Statement

This retrospective study was approved by the Ethics Committee of the hospital. Informed consent was waived due to the retrospective nature of the research and the use of de‐identified patient data. The study was conducted in accordance with the ethical principles outlined in the Declaration of Helsinki.

### 2.2 Enrolled Patients

To reduce selection bias inherent to retrospective studies, all eligible patients who received ICI monotherapy between June 2020 and June 2024 were consecutively included based on predefined clinical and laboratory criteria. All treatments were administered following national guidelines and standard clinical practice, enhancing the generalizability and real‐world applicability of the findings. First‐line monotherapy options included pembrolizumab, atezolizumab, or tislelizumab for tumors with PD‐L1 expression ≥ 50%, following standard dosing schedules: pembrolizumab 200 mg every 3 weeks, atezolizumab 1200 mg every 3 weeks, or tislelizumab 200 mg every 3 weeks. Second‐line monotherapy was offered following failure of first‐line chemoimmunotherapy, including pembrolizumab for tumors with PD‐L1 > 1% (200 mg every 3 weeks), or atezolizumab and tislelizumab regardless of PD‐L1 status, administered at their respective standard doses.

### 2.3 Inclusion and Exclusion Criteria

Eligible patients were aged ≥ 18 years with measurable disease per RECIST v1.1, assessed by CT within 1 month before starting immunotherapy, Eastern Cooperative Oncology Group (ECOG) performance status (PS) 0–2, and adequate liver and renal function (bilirubin, alkaline phosphatase, and transaminase < 1.5× upper normal limits; sodium > 125 mmol/L; normal calcium; creatinine clearance > 40 mL/min). Patients were enrolled irrespective of the completeness of baseline assessment data (e.g., questionnaires/scores) or subsequent treatment outcomes. Patients were excluded if they had active malignancies other than NSCLC, EGFR/ALK/ROS1 mutation‐positive NSCLC, active autoimmune diseases, thyroiditis, hypophysitis, acute cardiac failure, unstable angina, symptomatic brain metastases requiring high‐dose steroids, or seropositivity for hepatitis B, hepatitis C, or HIV. Pregnancy, lactation, cognitive impairment (Mini‐Mental State Exam ≤ 23) [17], and substance abuse disorders were also exclusion criteria.

### 2.4 Collection of Patient‐Reported Baseline Data Prior to ICIs Monotherapy

The data on nutritional status, psychological health, fatigue, and insomnia were originally documented in the medical records as part of routine clinical assessment prior to the initiation of ICI therapy. These data, recorded by trained clinical staff during initial consultations, were retrospectively extracted and analyzed for the purpose of this study. The Controlling Nutritional Status (CONUT) score assessed nutritional risk based on serum albumin, total cholesterol, and lymphocyte count, reflecting protein reserves, energy stores, and immune function. Scores range from 0 (no risk) to 12 (severe risk), with < 2 indicating low risk and ≥ 2 signifying higher nutritional risk or potential malnutrition [18]. Hope was measured using the 12‐item Herth Hope Index (HHI), which evaluates interconnectedness, positive readiness and expectation, and awareness of temporality and the future. Participants rated items on a 4‐point Likert scale (1 = “strongly disagree” to 4 = “strongly agree”), with total scores ranging from 12 to 48; higher scores reflect greater hope [14]. As no established cut‐off exists, the study used the median score of the enrolled patients for group stratification. Psychological distress, encompassing anxiety and depression, was assessed with the Hospital Anxiety and Depression Scale (HADS), a validated tool commonly used in cancer populations [19]. Each subscale includes seven items rated on a 4‐point scale, with scores > 7 indicating clinically significant anxiety or depression [20]. Fatigue was evaluated using the Brief Fatigue Inventory (BFI), a widely used measure for cancer‐related fatigue. The BFI comprises nine items: three on fatigue severity (0 = “no fatigue” to 10 = “worst imaginable fatigue”) and six on the impact of fatigue on daily activities (0 = “does not interfere” to 10 = “completely interferes”) [21]. Patients were categorized based on a global fatigue score cut‐off of ≥ 4 [22]. The Athens Insomnia Scale (AIS), an 8‐item self‐report questionnaire based on ICD‐10 criteria, assessed nocturnal sleep and daytime dysfunction. Scores range from 0 to 24, with higher scores indicating more severe insomnia; a cut‐off score of 6 was applied [16, 23].

### 2.5 Clinical Outcomes

The study evaluated treatment response, progression‐free survival (PFS), and overall survival (OS) in all patients starting from the initiation of ICI therapy. Tumor response was assessed according to RECIST 1.1 [24], with the first evaluation performed at week eight. Response categories included complete response (CR), partial response (PR), stable disease (SD), and progressive disease (PD). PFS was defined as the time from the start of ICI therapy to either the first documented disease progression or death from any cause, whichever occurred first. OS was defined as the time from treatment initiation to death from any cause. Patients without progression or death at the time of analysis were censored at their last follow‐up. All patients were followed until death or the end of the follow‐up period (October 30, 2024).

### 2.6 Statistical Analysis

Statistical analyses were performed using SPSS version 21.0 (IBM Corp., Armonk, NY) and GraphPad Prism version 8.0 (GraphPad Software, San Diego, CA). Continuous variables were expressed as mean ± standard deviation (SD) or median (interquartile range, IQR), depending on their distribution. Categorical variables were presented as counts and percentages. To evaluate the relationship between baseline characteristics (e.g., nutritional status, psychological health, fatigue, and insomnia) and immunotherapy response, Kruskal‐Wallis tests were applied for continuous variables, and post hoc comparisons were conducted using the Dunn‐Bonferroni correction. For survival analysis, Kaplan–Meier curves were generated to estimate OS and PFS, and differences between groups were assessed using the Log‐rank test. Prior to Cox regression modeling, multicollinearity among covariates was assessed using the variance inflation factor (VIF), with a VIF < 10 considered acceptable. Hazard ratios (HRs) and 95% confidence intervals (CIs) were calculated using Cox proportional hazards regression models. Statistical significance was set at *p <* 0.05 for all tests.

## 3 Results

### 3.1 Baseline Characteristics

The study enrolled 80 stage IV NSCLC patients (Table 1), predominantly male (66.3%); mean age 66.8 ± 9.0 years, BMI 20.1 ± 1.2 kg/m^2^, with adenocarcinoma as the major subtype (82.5%) and 43.8% having PD‐L1 ≥ 50%. ECOG scores were 0 (13.8%), 1 (52.5%), and 2 (33.8%). Among the 80 patients, 35 (43.8%) received ICI monotherapy as first‐line treatment, and 45 (56.3%) received ICI monotherapy in the second‐line setting. The median PFS was 10.5 months (mean 12.83 months; range 2–44 months), and the median OS was 20 months (mean 22.98 months; range 5–45 months). Baseline patient‐reported scores were as follows: CONUT (2.28 ± 1.89), HHI (39.68 ± 4.05), HADS‐Anxiety (4.06 ± 3.32), HADS‐Depression (5.71 ± 4.21), BFI (4.34 ± 1.53), and AIS (6.13 ± 3.38).

**TABLE 1:** Baseline characteristics of 80 advanced NSCLC patients undergoing immunotherapy.

Characteristic	Values
Age	66.79 ± 8.99 (39.00–84.00)
BMI	20.14 ± 1.22 (18.00–22.00)
Sex	
Male	53 (66.3%)
Female	27 (33.8%)
Histological subtype	
Squamous cell	14 (17.5%)
Adenocarcinoma	66 (82.5%)
Tumor PD‐L1 score	
≥ 50%	35 (43.8%)
< 50%	35 (43.8%)
Not evaluable	10 (12.5%)
ECOG‐performance status	
0	11 (13.8%)
1	42 (52.5%)
2	27 (33.8%)
Treatment	
First‐line (pembrolizumab, atezolizumab, or tislelizumab)	35 (43.8%)
Second‐line (atezolizumab or tislelizumab)	35 (43.8%)
Second‐line (pembrolizumab)	10 (12.5%)
Smoking status	
Never smoked	3 (3.8%)
Former smoker > 1 year	8 (10.0%)
Former smoker < 1 year	49 (61.3%)
Current smoker	20 (25.0%)
Patient‐reported baseline data	
CONUT	2.28 ± 1.89 (0.00–6.00)
HHI	39.68 ± 4.05 (29.00–48.00)
HADS‐A	4.06 ± 3.32 (0.00–14.00)
HADS‐D	5.71 ± 4.21 (0.00–14.00)
BFI	4.34 ± 1.53 (1.00–6.00)
AIS	6.13 ± 3.38 (1.00–12.00)

Abbreviations: AIS: Athens Insomnia Scale; BFI: Brief Fatigue Inventory; CONUT: Controlling Nutritional Status; HADS: Hospital Anxiety and Depression Scale; HHI: Herth Hope Index.

### 3.2 Pretreatment Nutritional Status, Psychological Health, Fatigue, and Insomnia Predict Response to ICIs in Advanced NSCLC

**FIGURE 1:**
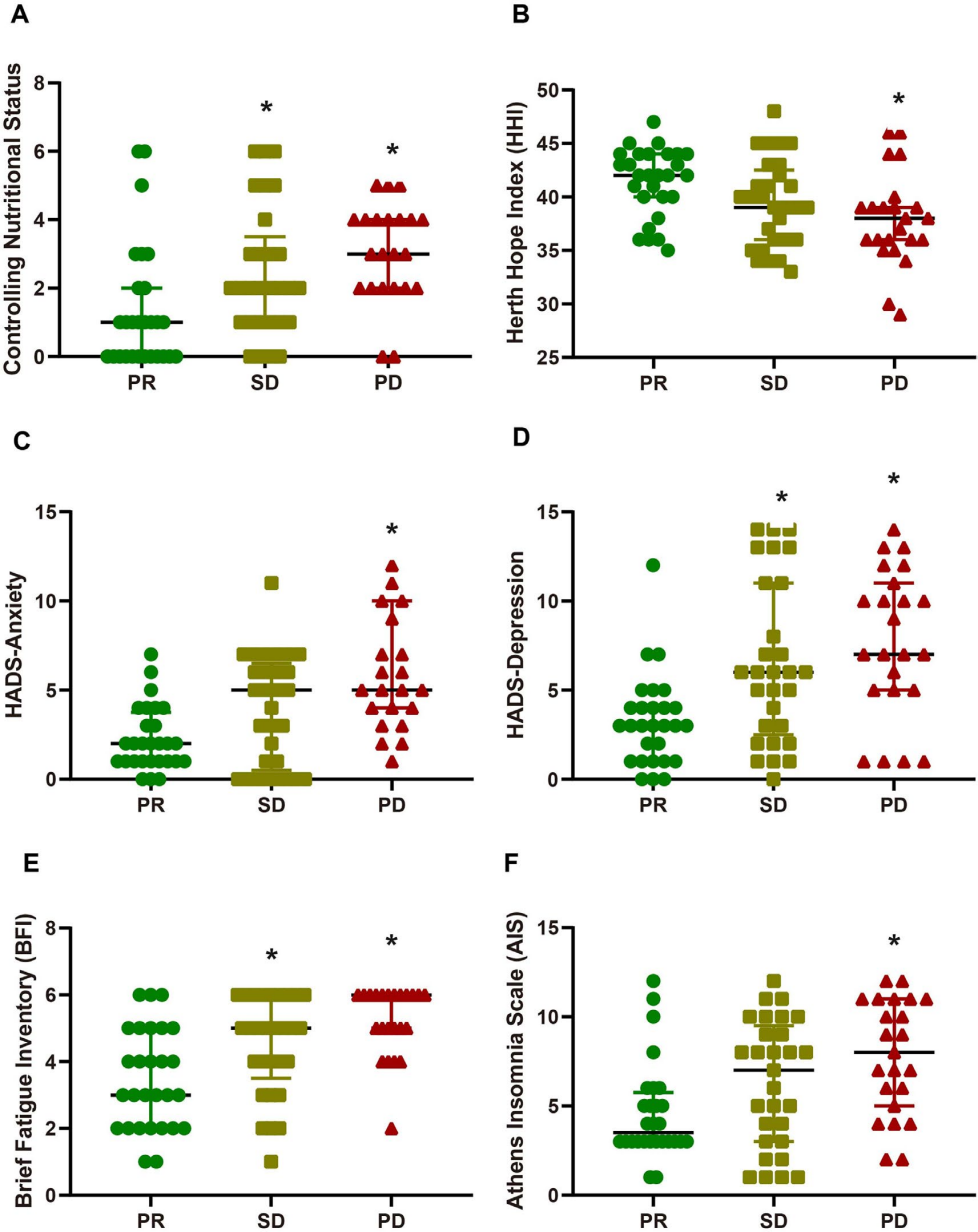
Pretreatment nutritional status, psychological health, fatigue, and insomnia associated with response to immune checkpoint inhibitor (ICI) monotherapy in advanced NSCLC. (A) Controlling Nutritional Status (CONUT) score; (B) Herth Hope Index (HHI); (C and D) Hospital Anxiety and Depression Score (HADS); (E) Brief Fatigue Inventory (BFI); (F) Athens Insomnia Scale (AIS); The clinical response of advanced non‐small cell lung cancer (NSCLC) patients treated with monotherapy immunotherapy was classified as follows: 28 patients achieved a partial response (PR); 29 patients had stable disease (SD); and 23 patients experienced progressive disease (PD). * indicates a significant difference compared to PD patients (*p <* 0.05).

Of 80 patients, 28 showed partial response (PR), 29 had stable disease (SD), and 30 experienced progressive disease (PD). Compared to PR patients (Figure 1), PD patients had significantly worse nutritional status (CONUT median 3 vs. 1; *p <* 0.05), lower hope (HHI median 38 vs. 42; *p <* 0.05), higher anxiety (HADS‐A median 5 vs. 2; *p <* 0.05), depression (HADS‐D median 7 vs. 3; *p <* 0.05), fatigue (BFI median 6 vs. 3; *p <* 0.05), and insomnia (AIS median 8 vs. 3.5; *p <* 0.05). SD patients showed intermediate scores, with significant differences in CONUT, depression, and fatigue compared to PR (all *p <* 0.05). These findings highlight the importance of pretreatment nutritional and psychosocial factors in predicting response to immunotherapy in advanced NSCLC.

### 3.3 Pretreatment Nutritional Status, Psychological Health, Fatigue, and Insomnia Predict Prognosis in Advanced NSCLC Treated With ICIs

**FIGURE 2:**
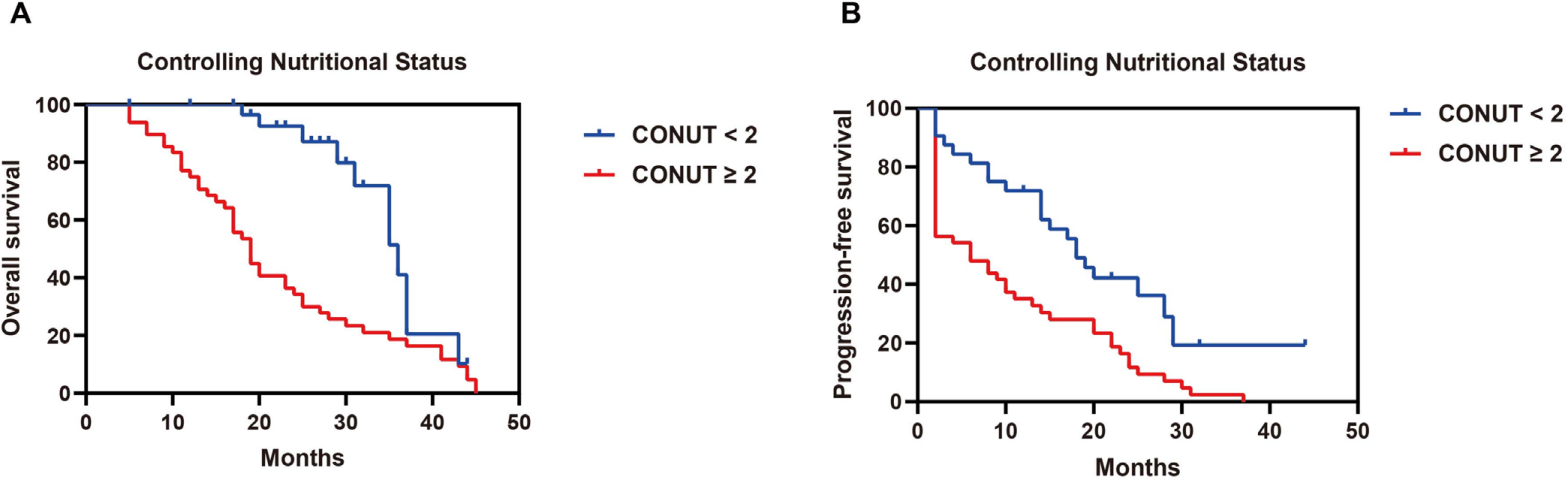
Pretreatment low nutritional risk associated with improved prognosis in advanced NSCLC received immune checkpoint inhibitor (ICI) monotherapy. Kaplan–Meier survival curves for overall survival (OS, A) and progression‐free survival (PFS, B) based on the Controlling Nutritional Status (CONUT) score. The CONUT score assesses nutritional status, with scores ranging from 0 (no risk) to 12 (severe risk). Patients were categorized into low‐risk (< 2, *n* = 32) and high‐risk (≥ 2, *n* = 48) groups.

**FIGURE 3:**
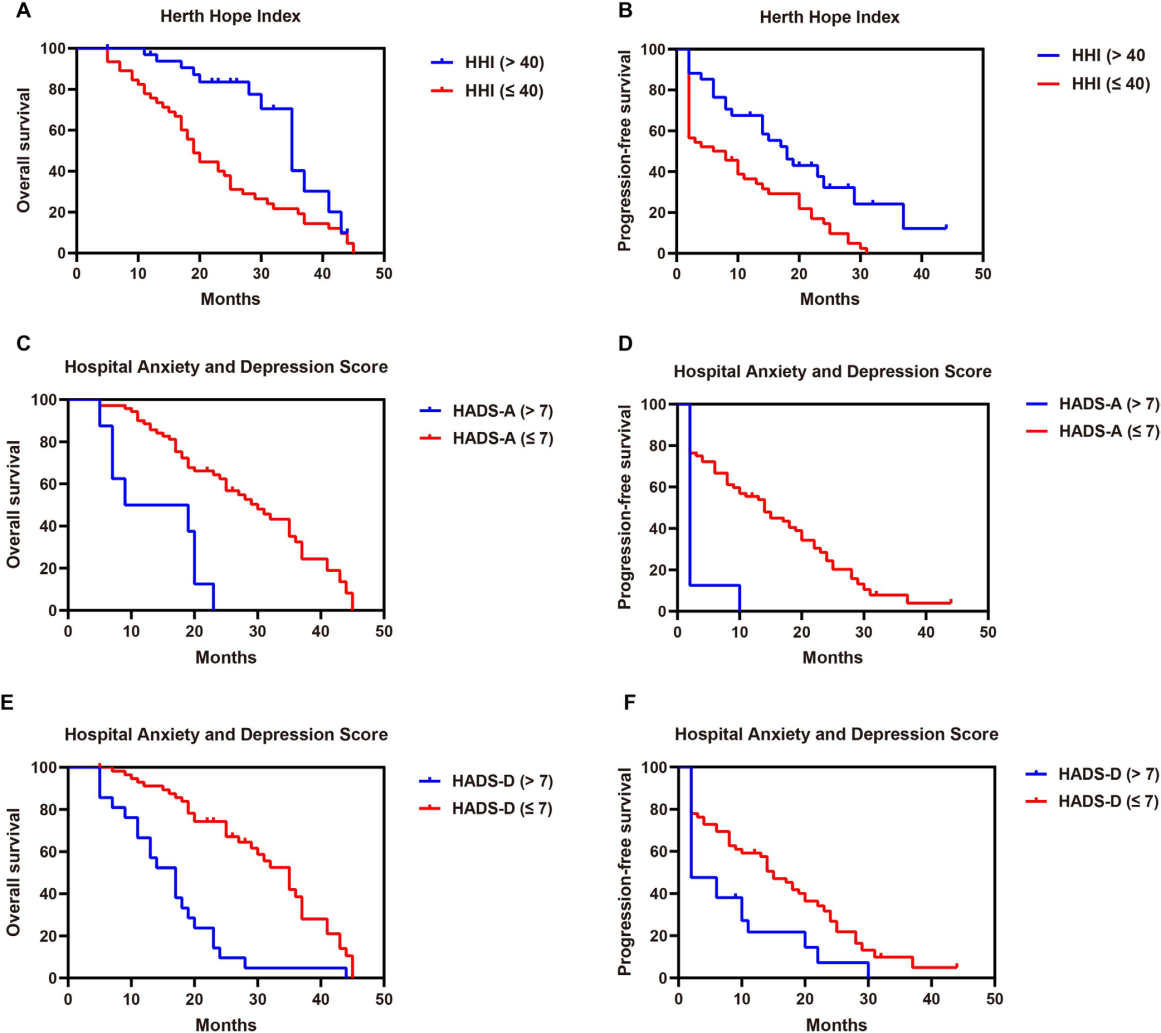
Pretreatment psychological health associated with prognosis in advanced NSCLC received immune checkpoint inhibitor (ICI) monotherapy. Kaplan–Meier survival curves for overall survival (OS, A, C, E) and progression‐free survival (PFS, B, D, F) based on pretreatment psychological health assessments; (A and B) Herth Hope Index (HHI) using a median cut‐off score of 40. Patients were grouped as HHI > 40 (*n* = 34) and HHI ≤ 40 (*n* = 46). (C and D) Hospital Anxiety and Depression Scale‐Anxiety (HADS‐A) with a cut‐off score of 7. Patients were categorized as HADS‐A > 7 (*n* = 8) and HADS‐A ≤ 7 (*n* = 72). (E and F) Hospital Anxiety and Depression Scale‐Depression (HADS‐D) with a cut‐off score of 7. Patients were grouped as HADS‐D > 7 (*n* = 21) and HADS‐D ≤ 7 (*n* = 59).

**FIGURE 4:**
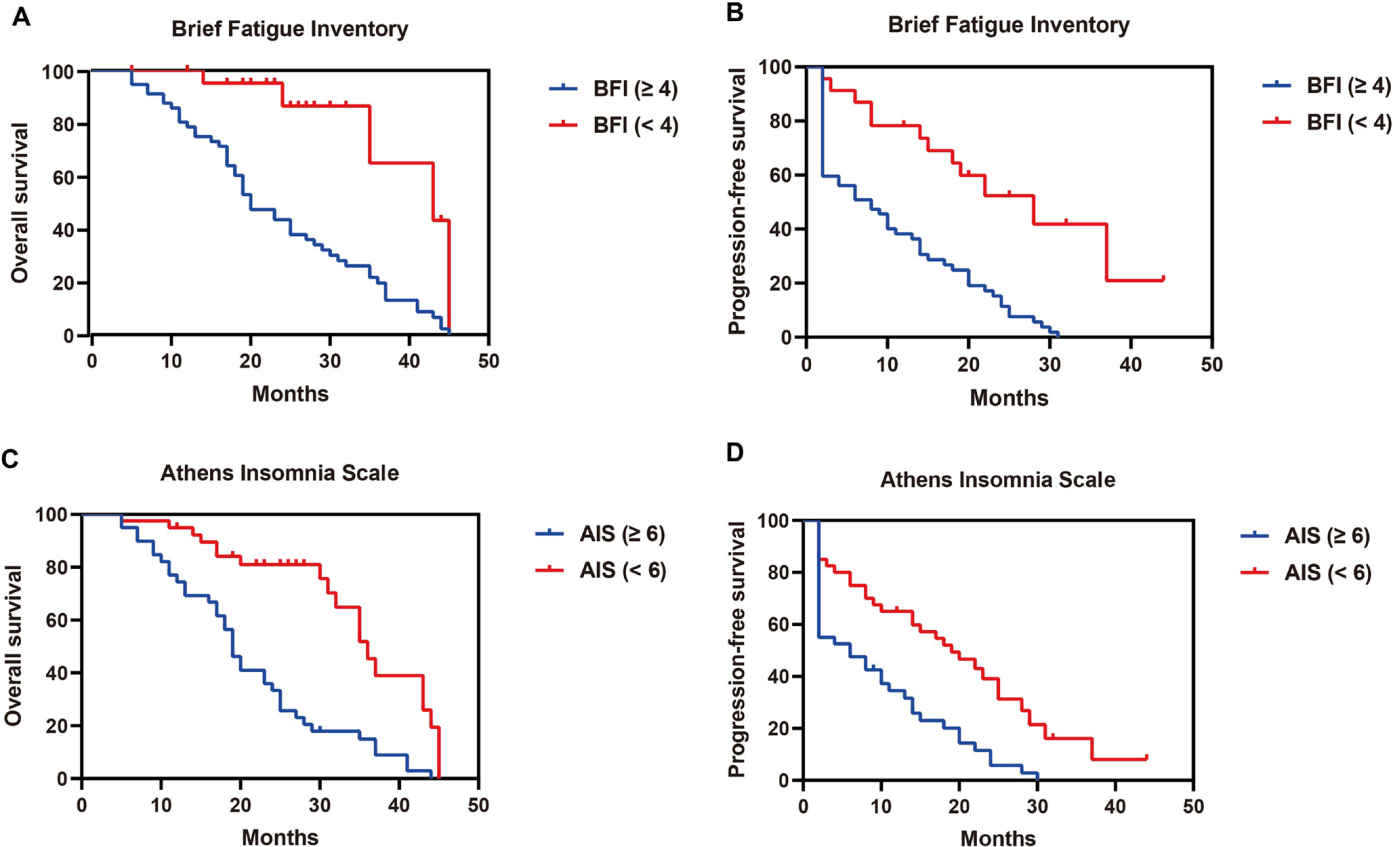
Pretreatment fatigue and insomnia associated with prognosis in advanced NSCLC receiving immune checkpoint inhibitor (ICI) monotherapy. Kaplan–Meier survival curves for overall survival (OS, A, C) and progression‐free survival (PFS, B, D) based on pretreatment fatigue and insomnia assessments. (A and B) Brief Fatigue Inventory (BFI) with a cut‐off score of 4. Patients were grouped as BFI ≥ 4 (*n* = 57) and BFI < 4 (*n* = 23). (C and D) Athens Insomnia Scale (AIS) with a cut‐off score of 6. Patients were categorized as AIS ≥ 6 (*n* = 40) and AIS < 6 (*n* = 40).

Low nutritional risk, higher hope, lower anxiety, lower depression, less fatigue, and milder insomnia significantly predicted improved survival in advanced NSCLC patients receiving ICIs. Patients with low nutritional risk showed longer median OS (36 vs. 19 months, *p <* 0.001) and PFS (18 vs. 6 months, *p =* 0.002) (Figure 2). Higher hope scores (HHI >40) were associated with prolonged OS (35 vs. 19 months, *p =* 0.001) and PFS (18 vs. 7 months, *p =* 0.002) (Figure 3A,B). Anxiety (HADS‐A > 7, Figure 3C,D) and depression (HADS‐D > 7, Figure 3E,F) significantly shortened OS (14 vs. 30 months and 17 vs. 35 months; both *p <* 0.001) and PFS (both 2 vs. 14 or 15 months; *p <* 0.001 and *p =* 0.007). Similarly, higher fatigue (BFI ≥ 4, Figure 4A,B) and severe insomnia (AIS ≥ 6, Figure 4C,D) significantly predicted worse OS (20 vs. 43 months and 19 vs. 36 months; both *p <* 0.001) and shorter PFS (8 vs. 28 months and 6 vs. 19 months; both *p <* 0.001).

### 3.4 Stratified Analysis of Pretreatment Nutritional Status, Psychological Health, Fatigue, and Insomnia Predicting Prognosis in Advanced NSCLC Patients Receiving ICIs According to Sex and Histology

As shown in Table S1, baseline factors significantly influenced survival with notable sex differences. In females, high CONUT scores (OS, *p =* 0.016; PFS, *p =* 0.008), low hope (OS, *p =* 0.006; PFS, *p =* 0.020), depression (OS, *p <* 0.001; PFS, *p =* 0.006), fatigue (OS, *p =* 0.008; PFS, *p =* 0.007), and insomnia (OS, *p =* 0.040; PFS, *p =* 0.012) predicted worse outcomes. In males, these factors had stronger associations, especially anxiety (OS, *p <* 0.001; PFS, *p =* 0.0002), depression (OS, *p <* 0.001; PFS, *p =* 0.029), fatigue (OS and PFS, *p =* 0.002), and insomnia (OS and PFS, *p =* 0.002). Moreover, these adverse effects were predominantly observed in adenocarcinoma patients (all *p <* 0.01), while in squamous cell carcinoma only anxiety (*p <* 0.001), depression (*p =* 0.005), and fatigue (PFS, *p =* 0.050) showed prognostic significance. This suggests greater sensitivity of adenocarcinoma patients to psychosocial and nutritional risks, emphasizing the need for targeted supportive care.

### 3.5 Cox Regression Analysis for PFS and OS in Advanced NSCLC Patients Receiving ICIs

Prior to conducting Cox regression analyses, multicollinearity among covariates was assessed using the VIF (data not shown). All variables exhibited acceptable VIF values below the commonly used threshold of 10, with most values under 3, indicating low to moderate collinearity. Although Treatment and TPS expression demonstrated slightly elevated VIFs (> 5), they remained within acceptable limits and were therefore retained for further analysis. Additionally, while some variables—such as CONUT, HHI, HADS‐A, HADS‐D, BFI, and AIS—may be conceptually interrelated due to their shared relevance to nutritional or psychosocial status, statistical assessment revealed no evidence of problematic collinearity. Thus, all candidate variables were included in the multivariate Cox models. As demonstrated in Tables 2 and 3, univariate Cox regression analysis identified several baseline factors significantly associated with both PFS and OS in advanced NSCLC patients receiving ICIs. Poor nutritional status, represented by higher CONUT scores (PFS: HR = 1.22, *p =* 0.002; OS: HR = 1.37, *p <* 0.001), and lower hope levels (HHI scores; PFS: HR = 0.91, *p =* 0.002; OS: HR = 0.87, *p <* 0.001) were significantly associated with worse outcomes. Psychological distress, including anxiety (HADS‐A; PFS: HR = 1.20, *p <* 0.001) and depression (HADS‐D; PFS: HR = 1.09, *p =* 0.002; OS: HR = 1.18, *p <* 0.001), as well as greater fatigue (BFI; PFS: HR = 1.45, *p <* 0.001; OS: HR = 1.62, *p <* 0.001) and insomnia severity (AIS; PFS: HR = 1.16, *p <* 0.001; OS: HR = 1.28, *p <* 0.001), were also significant predictors of reduced survival. Additionally, disease progression significantly correlated with poorer OS (HR = 4.55, *p =* 0.036). Significant factors from univariate analyses (*p <* 0.05) were included in multivariate Cox regression models. Multivariate analysis identified anxiety (HADS‐A; HR = 1.12, *p =* 0.015) as an independent predictor of shorter PFS, while depression (HADS‐D; HR = 1.09, *p =* 0.019) and fatigue (BFI; HR = 1.32, *p =* 0.033) remained independent predictors of reduced OS. These findings emphasize the clinical importance of pretreatment nutritional and psychosocial assessments to better predict prognosis in advanced NSCLC patients treated with ICIs.

**TABLE 2:** Univariate and multivariate Cox regression analysis of factors associated with progression‐free survival in advanced NSCLC patients undergoing immunotherapy.

Variable	Univariate analysis			Multivariate analysis		
	*p*	HR	95% CI	*p*	HR	95% CI
Age	0.528	1.011	0.977–1.046			
BMI	0.762	1.031	0.846–1.257			
Sex (male vs. female)	0.457	0.822	0.491–1.378			
Smoking						
Former smoker < 1 year versus never smoked	0.798	1.167	0.358–3.800			
Former smoker > 1 year versus never smoked	0.880	0.895	0.212–3.780			
Current smoker versus never smoked	0.456	1.591	0.469–5.400			
Histological (squamous vs. adenocarcinoma)	0.447	1.268	0.688–2.336			
Tumor PD‐L1 score						
< 50% versus not evaluable	0.646	1.195	0.558–2.561			
≥ 50% versus Not Evaluable	0.841	0.926	0.435–1.968			
Treatment (second line vs. first line)	0.400	1.233	0.757–2.007			
CONUT	**0.002**	**1.217**	**1.072–1.381**	0.621	0.954	0.792–1.150
HHI	**0.002**	**0.906**	**0.853–0.963**	0.957	0.998	0.916–1.087
HADS‐A	**< 0.001**	**1.195**	**1.109–1.287**	**0.015**	**1.122**	**1.023–1.230**
HADS‐D	**0.002**	**1.094**	**1.035–1.156**	0.486	1.026	0.954–1.104
BFI	**< 0.001**	**1.446**	**1.200–1.741**	0.061	1.244	0.990–1.559
AIS	**< 0.001**	**1.157**	**1.072–1.248**	0.313	1.055	0.948–1.169

*Note*: Significant predictors in the univariate analysis (*p <* 0.05) were included in the multivariate analysis. Bolded variables indicate statistical significance in the corresponding model.

Abbreviations: AIS: Athens Insomnia Scale; BFI: Brief Fatigue Inventory; CI: confidence interval; CONUT: Controlling Nutritional Status; HADS: Hospital Anxiety and Depression Scale; HHI: Herth Hope Index; HR: hazard ratio; TPS: tumor proportion score.

**TABLE 3:** Univariate and multivariate Cox regression analysis of factors associated with overall survival in advanced NSCLC patients undergoing immunotherapy.

Variable	Univariate analysis			Multivariate analysis		
	*p*	HR	95% CI	*p*	HR	95% CI
Age	0.528	1.011	0.977–1.046			
BMI	0.394	0.910	0.734–1.130			
Sex (male vs. female)	0.157	0.665	0.378–1.170			
Smoking						
Former smoker < 1 year versus never smoked	0.726	1.236	0.377–4.059			
Former smoker > 1 year versus never smoked	0.845	0.852	0.170–4.274			
Current smoker versus never smoked	0.568	1.440	0.411–5.043			
Histological (squamous vs. adenocarcinoma)	0.411	1.302	0.694–2.442			
Tumor PD‐L1 score						
< 50% versus not evaluable	0.716	0.861	0.385–1.927			
≥ 50% versus not evaluable	0.458	0.737	0.329–1.652			
Treatment (second line vs. first line)	0.481	1.210	0.712–2.055			
Disease progression	**0.036**	**4.547**	**1.105–18.709**	0.937	0.940	0.202–4.369
CONUT	**< 0.001**	**1.368**	**1.191–1.571**	0.707	1.036	0.863–1.242
HHI	**< 0.001**	**0.868**	**0.810–0.930**	0.800	0.989	0.905–1.080
HADS‐A	**< 0.001**	**1.239**	**1.134–1.353**	0.291	1.064	0.949–1.192
HADS‐D	**< 0.001**	**1.177**	**1.104–1.255**	**0.019**	**1.094**	**1.015–1.180**
BFI	**< 0.001**	**1.616**	**1.292–2.022**	**0.033**	**1.323**	**1.022–1.713**
AIS	**< 0.001**	**1.283**	**1.168–1.409**	0.094	1.112	0.982–1.259

*Note*: Significant predictors in the univariate analysis (*p <* 0.05) were included in the multivariate analysis. Bolded variables indicate statistical significance in the corresponding model.

Abbreviations: AIS: Athens Insomnia Scale; BFI: Brief Fatigue Inventory; CI: confidence interval; CONUT: Controlling Nutritional Status; HADS: Hospital Anxiety and Depression Scale; HHI: Herth Hope Index; HR: hazard ratio; TPS: tumor proportion score.

## 4 Discussion

This study emphasizes the critical influence of baseline nutritional status, psychological health, fatigue, and insomnia on clinical outcomes in advanced NSCLC patients undergoing ICIs monotherapy. Key findings revealed that higher nutritional risk (elevated CONUT scores), greater psychological distress (higher HADS‐A and HADS‐D scores), fatigue, and insomnia were associated with significantly worse PFS and OS. Notably, anxiety was an independent predictor of PFS, while depression and fatigue independently predicted OS. Conversely, higher levels of hope were strongly associated with improved survival outcomes, underscoring the prognostic value of both physiological and psychosocial factors.

Pretreatment nutritional status emerged as a pivotal determinant of survival. Patients with high nutritional risk had poorer PFS and OS, likely due to the interplay between malnutrition, systemic inflammation, and immune dysfunction. Prior studies corroborate the prognostic significance of the CONUT score in lung cancer. For instance, Onodera et al. linked high preoperative CONUT scores in resectable NSCLC to worse OS and recurrence‐free survival, mediated by adverse pathological features like pleural invasion and lung metastasis [11]. Similarly, Yilmaz et al. identified CONUT as an independent predictor of OS and PFS in small cell lung cancer [25]. Gul et al. found that poor nutritional status, as assessed by the CONUT and PNI scores, is a prevalent factor associated with significantly reduced overall survival in both NSCLC and SCLC patients with locally advanced and advanced‐stage lung cancer [26]. These findings suggest that early nutritional optimization, through dietary counseling or supplementation, may enhance the immune response and improve immunotherapy outcomes.

Advance NSCLC patients receiving immunotherapy or chemoimmunotherapy in clinical practice reported higher symptom prevalence [27]. Psychological health, particularly anxiety and depression, also played a significant role in survival outcomes. Elevated HADS‐A and HADS‐D scores were associated with poorer PFS and OS, respectively. Chronic psychological distress is known to dysregulate the hypothalamic–pituitary–adrenal axis and sympathetic nervous system, leading to elevated cortisol levels and pro‐inflammatory cytokines, which impair immune surveillance and tumor suppression [12, 28]. The findings of Gui et al. further highlight how psychological distress adversely impacts the tumor microenvironment and immune system, suggesting potential therapeutic targets [29]. Interventions such as cognitive‐behavioral therapy and pharmacological treatments could address psychological distress, positively influencing immunotherapy efficacy and survival outcomes.

Fatigue and insomnia were another significant predictor of worse survival outcomes. Hopkins et al. demonstrated that patient‐reported fatigue and global health outperform ECOG‐PS and LIPI in predicting OS, emphasizing the importance of incorporating symptom assessments into clinical care [30]. Addressing these symptoms through evidence‐based strategies, such as energy conservation techniques, physical therapy, or sleep hygiene education, could optimize treatment responses and improve quality of life. Lastly, hope emerged as a protective factor in this study. Patients with higher HHI scores exhibited significantly better PFS and OS, consistent with findings from Hsu et al., who identified hope as a modifiable factor influenced by fatigue and insomnia and treatment cycles [14]. Mechanistically, hope and psychological resilience may enhance immune competence, further improving survival. These insights support the integration of psychosocial interventions to bolster hope, alleviate distress, and optimize outcomes in NSCLC patients undergoing ICIs monotherapy.

This retrospective study has several limitations. First, the potential for selection bias and the relatively small sample size may restrict the generalizability of the findings. Second, the observational, single‐arm design precludes causal inference and limits the ability to definitively determine whether the identified prognostic factors, such as nutritional status, psychological health, fatigue, and insomnia, directly influence the efficacy of ICIs or represent general prognostic markers for advanced NSCLC. Thus, our results should be interpreted as hypothesis‐generating, highlighting potential prognostic associations rather than definitive predictive relationships. Third, detailed genetic profiling data (e.g., EGFR, KRAS, STK11, or KEAP1 mutations), which are known to influence ICI outcomes, were not available for all patients due to the retrospective nature of the study. As such, we were unable to explore potential interactions between genetic alterations and clinical or psychosocial variables. Fourth, although we adjusted for key clinical characteristics, unmeasured confounders, such as comorbidities, socioeconomic status, prior therapies, and concurrent medications, could have influenced immune function and treatment outcomes. Fifth, although several psychosocial and nutritional variables—such as CONUT, HHI, HADS‐A, HADS‐D, BFI, and AIS—may be conceptually interrelated, formal multicollinearity analysis using the VIF revealed no significant statistical collinearity. Nonetheless, the possibility of underlying conceptual overlap should be considered when interpreting the results. Future research should employ prospective designs, include comparator arms (e.g., chemotherapy‐treated patients), and account for a wider range of clinical, molecular, and psychosocial covariates to minimize residual confounding. Tailored interventions targeting these modifiable baseline factors may ultimately enhance survival and therapeutic benefit in patients with advanced NSCLC.

## 5 Conclusion

Baseline nutritional status, psychological health, fatigue, and insomnia were significantly associated with survival outcomes in advanced NSCLC patients receiving ICI monotherapy. Although our observational findings do not establish a causal relationship or confirm treatment‐specific predictive value, addressing these modifiable factors through targeted interventions could pave the way for more comprehensive and effective cancer care, improving both survival outcomes and quality of life.

## Conflicts of Interest

The authors declare no conflicts of interest.

## Supporting information


**Table S1:** Stratified analysis of baseline factors predicting prognosis in advanced NSCLC patients receiving ICIs according to sex and histology.

## Data Availability

The data that support the findings of this study are available from the corresponding author upon reasonable request.

## References

[1] P. L. Su , K. Chakravarthy , N. Furuya , et al., “DLL3‐Guided Therapies in Small‐Cell Lung Cancer: From Antibody‐Drug Conjugate to Precision Immunotherapy and Radioimmunotherapy,” Molecular Cancer 23, no. 1 (2024): 97. 38730427 10.1186/s12943-024-02012-z PMC11084107

[2] A. Lahiri , A. Maji , P. D. Potdar , et al., “Lung Cancer Immunotherapy: Progress, Pitfalls, and Promises,” Molecular Cancer 22, no. 1 (2023): 40. 36810079 10.1186/s12943-023-01740-y PMC9942077

[3] K. Suresh , J. Naidoo , C. T. Lin , and S. Danoff , “Immune Checkpoint Immunotherapy for Non‐Small Cell Lung Cancer: Benefits and Pulmonary Toxicities,” Chest 154, no. 6 (2018): 1416–1423. 30189190 10.1016/j.chest.2018.08.1048 PMC6335259

[4] S. Tang , C. Qin , H. Hu , et al., “Immune Checkpoint Inhibitors in Non‐Small Cell Lung Cancer: Progress, Challenges, and Prospects,” Cells 11, no. 3 (2022): 320. 35159131 10.3390/cells11030320 PMC8834198

[5] M. Boussageon , A. Swalduz , C. Chouaid , and O. Bylicki , “First‐Line Treatment of Advanced Non‐Small‐Cell Lung Cancer With Immune‐Checkpoint Inhibitors: New Combinations and Long‐Term Data,” BioDrugs 36, no. 2 (2022): 137–151. 35147894 10.1007/s40259-022-00515-z

[6] N. Krzyzanowska , P. Krawczyk , K. Wojas‐Krawczyk , T. Kucharczyk , and J. Milanowski , “Immunotherapy in Non‐Small‐Cell Lung Cancer Patients With Driver Alterations: A New Strategy?,” Cells 11, no. 20 (2022): 3280. 36291146 10.3390/cells11203280 PMC9600960

[7] C. Madeddu , S. Busquets , C. Donisi , et al., “Effect of Cancer‐Related Cachexia and Associated Changes in Nutritional Status, Inflammatory Status, and Muscle Mass on Immunotherapy Efficacy and Survival in Patients With Advanced Non‐Small Cell Lung Cancer,” Cancers (Basel) 15, no. 4 (2023): 1076. 36831431 10.3390/cancers15041076 PMC9953791

[8] M. MacDonald , D. Poei , A. Leyba , et al., “Real World Prognostic Utility of Platelet Lymphocyte Ratio and Nutritional Status in First‐Line Immunotherapy Response in Stage IV Non‐Small Cell Lung Cancer,” Cancer Treatment and Research Communications 36 (2023): 100752. 37611343 10.1016/j.ctarc.2023.100752 PMC11160511

[9] H. Yilmaz , B. Nigdelioglu , E. Oktay , and N. Meydan , “Clinical Significance of Postoperatif Controlling Nutritional Status (CONUT) Score in Glioblastoma Multiforme,” Journal of Clinical Neuroscience 86 (2021): 260–266. 33775339 10.1016/j.jocn.2021.01.036

[10] M. N. Sheas , S. R. Ali , W. Safdar , et al., “Nutritional Assessment in Cancer Patients,” Cancer Treatment and Research 185 (2023): 285–310. 37306914 10.1007/978-3-031-27156-4_14

[11] K. Onodera , H. Notsuda , T. Watanabe , et al., “The CONUT Score Is Associated With the Pathologic Grade in Non‐Small Cell Lung Cancer,” Surgery Today 54, no. 12 (2024): 1437–1444. 38709286 10.1007/s00595-024-02860-8 PMC11582223

[12] M. G. Oyola and R. J. Handa , “Hypothalamic‐Pituitary‐Adrenal and Hypothalamic‐Pituitary‐Gonadal Axes: Sex Differences in Regulation of Stress Responsivity,” Stress 20, no. 5 (2017): 476–494. 28859530 10.1080/10253890.2017.1369523 PMC5815295

[13] M. G. Cohen , A. D. Althouse , R. M. Arnold , et al., “Hope and Advance Care Planning in Advanced Cancer: Is There a Relationship?,” Cancer 128, no. 6 (2022): 1339–1345. 34787930 10.1002/cncr.34034 PMC8882158

[14] C. C. Hsu , Y. H. Lee , M. R. Chen , et al., “Hope and Its Relationship With Treatment/Physical Related Factors in Lung Cancer Patients Receiving Immunotherapy,” Journal of the Formosan Medical Association 24, no. 7 (2024): 660–665. 10.1016/j.jfma.2024.06.023 38971711

[15] A. Buttner‐Teleaga , Y. T. Kim , T. Osel , and K. Richter , “Sleep Disorders in Cancer: A Systematic Review,” International Journal of Environmental Research and Public Health 18, no. 21 (2021): 11696. 34770209 10.3390/ijerph182111696 PMC8583058

[16] C. Y. Lin , A. S. K. Cheng , B. Nejati , et al., “A Thorough Psychometric Comparison Between Athens Insomnia Scale and Insomnia Severity Index Among Patients With Advanced Cancer,” Journal of Sleep Research 29, no. 1 (2020): e12891. 31328319 10.1111/jsr.12891

[17] M. F. Folstein , S. E. Folstein , and P. R. McHugh , “Mini‐Mental State. A Practical Method for Grading the Cognitive State of Patients for the Clinician,” Journal of Psychiatric Research 12, no. 3 (1975): 189–198. 1202204 10.1016/0022-3956(75)90026-6

[18] L. Chen , H. Sun , R. Zhao , et al., “Controlling Nutritional Status (CONUT) Predicts Survival in Gastric Cancer Patients With Immune Checkpoint Inhibitor (PD‐1/PD‐L1) Outcomes,” Frontiers in Pharmacology 13 (2022): 836958. 35308215 10.3389/fphar.2022.836958 PMC8931544

[19] M. A. Annunziata , B. Muzzatti , E. Bidoli , et al., “Hospital Anxiety and Depression Scale (HADS) Accuracy in Cancer Patients,” Support Care Cancer 28, no. 8 (2020): 3921–3926. 31858249 10.1007/s00520-019-05244-8

[20] K. B. Gordon , A. W. Armstrong , C. Han , et al., “Anxiety and Depression in Patients With Moderate‐To‐Severe Psoriasis and Comparison of Change From Baseline After Treatment With Guselkumab vs. Adalimumab: Results From the Phase 3 VOYAGE 2 Study,” Journal of the European Academy of Dermatology and Venereology 32, no. 11 (2018): 1940–1949. 29706008 10.1111/jdv.15012

[21] C. H. Shih , P. C. Chou , J. H. Chen , et al., “Cancer‐Related Fatigue Classification Based on Heart Rate Variability Signals From Wearables,” Frontiers in Medicine (Lausanne) 10 (2023): 1103979. 10.3389/fmed.2023.1103979 PMC10169588 37181354

[22] H. Poort , J. M. Jacobs , W. F. Pirl , J. S. Temel , and J. A. Greer , “Fatigue in Patients on Oral Targeted or Chemotherapy for Cancer and Associations With Anxiety, Depression, and Quality of Life,” Palliative & Supportive Care 18, no. 2 (2020): 141–147. 31535613 10.1017/S147895151900066X PMC7489872

[23] M. A. Mafla‐Espana , M. D. Torregrosa , M. Beamud‐Cortes , L. Bermell‐Marco , J. Rubio‐Briones , and O. Cauli , “Comparison of Frailty Criteria, Cognitive Function, Depressive and Insomnia Symptoms in Men With Localized and Advanced Prostate Cancer Under Androgen Deprivation Therapy,” Healthcare (Basel) 11, no. 9 (2023): 1266. 37174808 10.3390/healthcare11091266 PMC10178148

[24] E. A. Eisenhauer , P. Therasse , J. Bogaerts , et al., “New Response Evaluation Criteria in Solid Tumours: Revised RECIST Guideline (Version 1.1),” European Journal of Cancer 45, no. 2 (2009): 228–247. 19097774 10.1016/j.ejca.2008.10.026

[25] A. Yilmaz , S. B. Tekin , M. Bilici , and H. Yilmaz , “The Significance of Controlling Nutritional Status (CONUT) Score as a Novel Prognostic Parameter in Small Cell Lung Cancer,” Lung 198, no. 4 (2020): 695–704. 32424800 10.1007/s00408-020-00361-2

[26] B. Gul , S. Metintas , G. Ak , S. Yilmaz , and M. Metintas , “The Relationship Between Nutritional Status and Prognosis in Patients With Locally Advanced and Advanced Stage Lung Cancer,” Support Care Cancer 29, no. 6 (2021): 3357–3365. 33128137 10.1007/s00520-020-05856-5

[27] L. E. Steffen McLouth , T. W. Lycan , B. J. Levine , et al., “Patient‐Reported Outcomes From Patients Receiving Immunotherapy or Chemoimmunotherapy for Metastatic Non‐Small‐Cell Lung Cancer in Clinical Practice,” Clinical Lung Cancer 21, no. 3 (2020): 255–263. 31917067 10.1016/j.cllc.2019.11.015 PMC7730068

[28] P. Jerem and L. M. Romero , “It's Cool to Be Stressed: Body Surface Temperatures Track Sympathetic Nervous System Activation During Acute Stress,” Journal of Experimental Biology 226, no. 20 (2023): jeb246552. 37767773 10.1242/jeb.246552 PMC10629684

[29] H. Gui , X. Chen , L. Li , et al., “Psychological Distress Influences Lung Cancer: Advances and Perspectives on the Immune System and Immunotherapy,” International Immunopharmacology 121 (2023): 110251. 37348230 10.1016/j.intimp.2023.110251

[30] A. M. Hopkins , J. Wagner , G. Kichenadasse , N. Modi , A. Rowland , and M. J. Sorich , “Patient‐Reported Outcomes as a Prognostic Marker of Survival in Patients With Advanced Nonsmall Cell Lung Cancer Treated With Immunotherapy,” International Journal of Cancer 147, no. 11 (2020): 3085–3089. 32492185 10.1002/ijc.33133

