# Supplementary material for: Impact of Baseline Nutritional Status, Psychological Health, Fatigue, and Insomnia on Outcomes of Immune Checkpoint Inhibitors in Advanced Non‐Small Cell Lung Cancer: A Retrospective Cohort Study
Source: Kaohsiung J Med Sci. 2025 Aug 24;42(2):e70097. doi: 10.1002/kjm2.70097 (PMC12884818; doi:10.1002/kjm2.70097)
Supplement: Supplementary file 1 — Table S1: Stratified analysis of baseline factors predicting prognosis in advanced NSCLC patients receiving ICIs according to sex and histology. [file KJM2-42-e70097-s001.docx]

**Supplementary Table 1** Stratified analysis of baseline factors predicting prognosis in advanced NSCLC patients receiving ICIs according to sex and histology

| **Factor** | **Sex** | **Outcome** | **Median_0** | **Median_1** | ***χ*^2^** | ***P*** | **Histological Subtype** | **Outcome** | **Median_0** | **Median_1** | ***χ*^2^** | ***P*** |
| --- | --- | --- | --- | --- | --- | --- | --- | --- | --- | --- | --- | --- |
| CONUT_cat | Female | OS | 36 | 17 | 5.83 | 0.016 | Adenocarcinoma | OS | 37 | 19 | 15.28 | 9.26E-05 |
|  |  | PFS | 14 | 2 | 6.93 | 0.008 |  | PFS | 18 | 6 | 10.57 | 0.001 |
| HHI_cat |  | OS | 17 | 37 | 7.56 | 0.006 |  | OS | 20 | 41 | 14.61 | 1.32E-04 |
|  |  | PFS | 4 | 14 | 5.42 | 0.020 |  | PFS | 8 | 19 | 10.2 | 0.001 |
| HADS_A_cat |  | OS | 29 | 20 | 0.2 | 0.653 |  | OS | 31 | 19 | 14.04 | 1.79E-04 |
|  |  | PFS | 10 | 2 | 2 | 0.157 |  | PFS | 14 | 2 | 14.01 | 1.82E-04 |
| HADS_D_cat |  | OS | 31 | 17 | 12.33 | 4.46E-04 |  | OS | 37 | 17 | 25.63 | 4.13E-07 |
|  |  | PFS | 14 | 2 | 7.63 | 0.006 |  | PFS | 15 | 2 | 9.61 | 0.002 |
| BFI_cat |  | OS | inf | 19 | 7.06 | 0.008 |  | OS | 45 | 23 | 16.03 | 6.25E-05 |
|  |  | PFS | inf | 4 | 7.36 | 0.007 |  | PFS | 28 | 9 | 14.06 | 1.77E-04 |
| AIS_cat |  | OS | 36 | 18 | 4.24 | 0.040 |  | OS | 44 | 19 | 22.91 | 1.70E-06 |
|  |  | PFS | 14 | 4 | 6.34 | 0.012 |  | PFS | 22 | 6 | 14.66 | 1.29E-04 |
| CONUT_cat | Male | OS | 37 | 23 | 9.89 | 0.002 | Squamous Cell | OS | 35 | 18 | 3.42 | 0.064 |
|  |  | PFS | 19 | 8 | 5.59 | 0.018 |  | PFS | 20 | 2 | 1.2 | 0.273 |
| HHI_cat |  | OS | 23 | 35 | 7.19 | 0.007 |  | OS | 18 | 35 | 0.47 | 0.494 |
|  |  | PFS | 8 | 18 | 5.2 | 0.023 |  | PFS | 2 | 8 | 0.63 | 0.426 |
| HADS_A_cat |  | OS | 35 | 9 | 22.1 | 2.59E-06 |  | OS | 25 | 7 | 13 | 3.11E-04 |
|  |  | PFS | 17 | 2 | 13.68 | 2.16E-04 |  | PFS | 8 | 2 | 1.33 | 2.48E-01 |
| HADS_D_cat |  | OS | 35 | 17 | 16.77 | 4.22E-05 |  | OS | 25 | 7 | 7.83 | 5.13E-03 |
|  |  | PFS | 18 | 6 | 4.79 | 0.029 |  | PFS | 8 | 2 | 1.85 | 0.174 |
| BFI_cat |  | OS | 43 | 23 | 9.97 | 0.002 |  | OS | 35 | 19 | 2.55 | 0.110 |
|  |  | PFS | 22 | 8 | 10.02 | 0.002 |  | PFS | 37 | 2 | 3.83 | 0.050 |
| AIS_cat |  | OS | 43 | 23 | 14.73 | 1.24E-04 |  | OS | 35 | 18 | 3.39 | 6.57E-02 |
|  |  | PFS | 20 | 8 | 9.21 | 0.002 |  | PFS | 8 | 2 | 3.29 | 0.069 |

Note: The "_cat" suffix represents categorical variables dichotomized as follows: CONUT_cat: 0 = low-risk (CONUT < 2), 1 = high-risk (CONUT ≥ 2); HHI_cat: 0 = low hope (HHI ≤ 40), 1 = high hope (HHI > 40); HADS_A_cat: 0 = low anxiety (HADS-A ≤ 7), 1 = high anxiety (HADS-A > 7); HADS_D_cat: 0 = low depression (HADS-D ≤ 7), 1 = high depression (HADS-D > 7); BFI_cat: 0 = mild fatigue (BFI < 4), 1 = moderate-to-severe fatigue (BFI ≥ 4); AIS_cat: 0 = mild insomnia (AIS < 6), 1 = moderate-to-severe insomnia (AIS ≥ 6). Median_0 and Median_1 represent the median survival time (in months) for patients in categories 0 and 1, respectively. Survival differences were evaluated using the log-rank test. Abbreviations: OS = overall survival; PFS = progression-free survival; NSCLC = non-small cell lung cancer; CONUT = Controlling Nutritional Status; HHI = Herth Hope Index; HADS = Hospital Anxiety and Depression Scale; BFI = Brief Fatigue Inventory; AIS = Athens Insomnia Scale.
